# Toward *In Situ* Monitoring of Water Contamination by Nitroenergetic Compounds

**DOI:** 10.3390/s121114953

**Published:** 2012-11-06

**Authors:** Brandy J. Johnson, Iwona A. Leska, Alejandro Medina, Norris F. Dyson, Mansoor Nasir, Brian J. Melde, Jenna R. Taft, Paul T. Charles

**Affiliations:** 1 Center for Bio/Molecular Science and Engineering, Naval Research Laboratory, Washington, DC 20375, USA; E-Mails: brian.melde@nrl.navy.mil (B.J.M.); paul.charles@nrl.navy.mil (P.T.C.); 2 NOVA Research Incorporated, Alexandria, VA 22308, USA; E-Mails: iwona.leska.ctr@nrl.navy.mil (I.A.L.); jennartaft@gmail.com (J.R.T.); 3 Department of Chemistry, University of Puerto Rico at Arecibo, Arecibo 00613, Puerto Rico; E-Mail: anidem-ale@hotmail.com; 4 College of Engineering, North Carolina A&T State University, Greensboro, NC 27411, USA; E-Mail: nfdyson@ncat.edu; 5 Biomedical Engineering, Lawrence Technological University, Southfield, MI 48075, USA; E-Mail: mnasir@ltu.edu

**Keywords:** perchlorate, RDX, 2,4,6-trinitrotoluene, periodic mesoporous organosilica, electrochemical, solid phase extraction

## Abstract

We have previously described the application of novel porous organosilicate materials to the preconcentration of nitroenergetic targets from aqueous solution prior to HPLC analysis. The performance of the sorbents and the advantages of these types of materials over commercially available solid phase extraction sorbents have been demonstrated. Here, the development of systems for application of those sorbents to *in situ* monitoring is described. Considerations such as column pressure, particulate filtration, and component durability are discussed. The diameter of selected column housings, the sorbent bed depth, and the frits utilized significantly impact the utility of the sorbent columns in the prototype system. The impact of and necessity for improvements in the morphological characteristics of the sorbents as they relate to reduction in column pressure are detailed. The results of experiments utilizing a prototype system are presented. Data demonstrating feasibility for use of the sorbents in preconcentration prior to ion mobility spectrometry is also presented.

## 1. Introduction

Contamination of water sources at sites associated with U.S. Department of Defense (DoD) activities is an ongoing concern. Compounds such as nitroenergetics and perchlorates, used as components of common ordnance, are of particular interest. Many nitroenergetics are known carcinogens or suspected cancer causing agents [1]. In addition to the potential health hazard posed to military personnel and their families residing on DoD installations, the migration and leaching of these carcinogens to the surrounding population, agricultural regions, and neighboring wildlife is a serious concern [2,3]. Contamination by these compounds results from testing, training, and disposal of ordnance with device malfunction being the most common cause of residual contamination. Contamination can also result from spillage during manufacture of ordnance. Contamination from munitions left in the environment, such as those disposed of by burying or unrecovered land mines, also presents an issue for non-DoD sites [4].

Monitoring at sites of potential contamination is an ongoing task and can present significant challenges. The rapid diffusion and migration of nitroenergetic compounds and their resulting dilution leads to low concentration levels in collected samples. Due to the low concentrations, matrix complexity, and strict reproducibility and reliability constraints, offsite analysis of samples is the standard for evaluating sites of interest [5]. This type of sample collection and analysis process is both expensive and time consuming. The methods employed utilize liquid or gas chromatography, techniques that do not lend themselves well to portable devices and methods. Onsite methods are desirable as indicators of the need for further testing and/or *in situ* methods for continuous monitoring of contamination levels. These approaches are generally less time consuming and can be considerably less expensive. Unfortunately, many portable methods either lack the robustness, ease of use, quantitative capability, or sensitivity necessary for field application [6–9].

Electrochemical (EC) detection shows promise for onsite monitoring applications offering the potential for low-cost, low-power solutions. Remote monitoring systems based on electrochemical measurements have been described [10]. Sensitivity to matrix components and interferents has limited the applicability of this technique. Another promising technology for field application as miniaturized sensors is ion mobility spectroscopy (IMS) [11,12]. Several hand-held instruments based on IMS are available [13]. The technology has also been applied to monitoring concentrations of contaminants in soils [14]. Current applications are limited to compounds with higher vapor pressures or to samples that are pre-heated due to the need for samples to be gaseous.

Standardized methods developed for field applications typically rely on preconcentration of targets to achieve concentrations within the range of analysis. The procedure outlined by the U.S. Environmental Protection Agency (EPA) for colorimetric detection of TNT and RDX, for example, relies on adsorption of target from two liters of sample solution onto functionalized membranes [15,16]. This type of solid phase extraction (SPE) of targets offers the potential to address the shortfalls in both EC and IMS based detection. SPE involves adsorption of target onto a solid support. Desorption is accomplished through the use of a thermal process or through elution under particular conditions. The intent is to adsorb target from a large sample volume and desorb it into a small volume leading to a higher target concentration. Adsorption by the SPE material may be through specific, semi-selective, or non-selective interactions. A wide range of approaches to the generation of these materials have been described for application to differing targets and matrices [17–21].

We have previously described our efforts focused on the development of organosilicate materials for preconcentration of nitroenergetic compounds [22–24]. The sorbents combine semi-selective binding with the rugged character of a silicate material to provide a sorbent applicable under a range of conditions. Advances in the synthesis of these materials provided the potential for improving both morphological and binding characteristics. The materials were shown to provide preconcentration of nitroenergetic targets from a range of matrices including soil extracts [25]. The organosilicate sorbents were also found to recover a greater percentage of targets than commercially available materials intended for these applications and were less sensitive to variations in matrix composition. Sorbents were also developed for the capture of organophosphate and solvent targets [26–28]. Here, we discuss the development of systems for the application of these sorbents with a view toward their combination with electrochemical and ion mobility spectrometry based detection. We also describe the impact of and necessity for alterations in the morphological characteristics of the SPE sorbents when applied with these systems.

## 2. Experimental Section

Sample solutions of 2,4,6-trinitrotoluene (TNT), 2,4-dinitrotoluene (DNT), nitroglycerine (NG), 1,3,5-trinitro-1,3,5-triazacyclohexane (RDX), and octahydro-1,3,5,7-tetranitro-1,3,5,7-tetrazocane (HMX) were prepared by dilution of 1 mg/mL reference standards in acetonitrile obtained from Cerilliant (Round Rock, TX, USA) in the appropriate matrix. Chemicals were used as received. Water was deionized to 18.2 MΩ cm using a Millipore Milli-Q UV-Plus water purification system. Ground water samples were collected from household wells in Clarksville, MD (depth of 114 m) and Fulton, MD (depths of 122 and 213 m), USA.

Column breakthrough experiments were completed using the indicated sorbent material in a BioRad disposable polypropylene column. Added volumes were collected individually for subsequent analysis. All samples were filtered using 0.2 μm polytetrafluoroethylene (PTFE) syringe filters prior to analysis. Analysis of the various volumes containing nitroenergetic targets was accomplished on a Shimadzu High Performance Liquid Chromatography (HPLC) system with dual-plunger parallel flow solvent delivery modules (LC-20AD) and an auto-sampler (SIL-20AC) coupled to a photodiode array detector (SPD-M20A). A modification of EPA method 8330 was employed. The stationary phase was a 250 × 4.6 mm Waters Symmetry C18 (5 μm) analytical column; an isocratic 50:50 methanol:water mobile phase was employed. A 100 μL sample injection was used with a flow rate of 0.9 mL/min. UV/vis detection of targets was accomplished at 254 nm with the exception of nitroglycerin which was detected at 214 nm. This method gives reliable detection at 8 ppb for the targets considered. Eight point target calibration curves were used with all experiments to verify method performance, and stock target concentrations were measured as a reference for each experiment. The variation in the calibration curves was ±5%.

Studies focused on development of the sorbent materials utilized in these studies have been published previously [23,24,29]. Bis(trimethoxysilylethyl)benzene (DEB, mixture of 1,4- and 1,3-isomers) was obtained from Gelest, Inc. (Morrisville, PA, USA). 1,2-bis(Trimethoxysilyl)ethane 96% (BTME), tetramethyl orthosilicate 98% (TMOS), hydrochloric acid 37% (HCl), nitric acid 70% (HNO_3_), mesitylene (1,3,5-trimethylbenzene, TMB), dichloromethane ≥ 99.5%, 3,5-dinitrobenzoyl chloride ≥ 98%, and magnesium turnings were obtained from Sigma-Aldrich (St. Louis, MO, USA). Ethanol (200 proof) was obtained from the Warner-Graham Company (Cockeysville, MD, USA). Sodium bicarbonate (NaHCO_3_) was purchased from Fisher (Hampton, NH, USA). Pluronic^®^ P123 (P123) was donated generously by BASF (can be purchased from Sigma-Aldrich as poly(ethylene glycol)-*block*-poly(propylene glycol)-*block*-poly(ethylene glycol) average M_n_ ∼ 5,800).

For synthesis of MM1 and ED13 sorbents, the imprint template was first synthesized by esterification of P123 with 3,5-dinitrobenzoyl chloride. P123 (8 g), 3,5-dinitrobenzoyl chloride (1.27 g), and magnesium turnings were added to 60 mL of dichloromethane and refluxed for 2 h. The solution was shaken with 60 mL of 2% aqueous NaHCO_3_, and the organic phase was collected and evaporated to obtain the yellow product, DNB-imprint P123.

To prepare the MM1 sorbent, 1.66 g of P123 and 0.24 g of DNB-imprint P123 were dissolved with 0.2 g of TMB in 6.07 g of 0.1 M HNO_3_ with magnetic stirring and heating ca. 60 °C. The stirring mixture was allowed to cool to room temperature and a silane mixture of 1.06 g BTME and 1.468 g DEB (0.00784 mol total bis-silane with BTME: DEB ratio = 1) was added drop-wise. The mixture was stirred until homogeneous, transferred to a culture tube, sealed tightly, and heated at 60 °C overnight (≥18 h). A white gel formed during this initial heating and the culture tube was unsealed to dry for 2 d at 60 °C, then 2 d at 80 °C. The monolithic material was refluxed in 1 M HCl in ethanol for at least 12 h three times to extract P123, a process that often broke the material down to a powder. It was collected by vacuum filtration, washed with ethanol and water, and dried at 110 °C. The ED13 sorbent was prepared following the same procedure with the exceptions that 0.6 g TMB and 7.5 g 0.1 M HNO_3_ were utilized. The D7 sorbent utilized 0.9 g TMB and 7.5 g 0.1 M HNO_3_. In addition, for D7 the 0.00784 mol total bis-silane consisted exclusively of DEB, and 1.9 g P123 was used with no imprint template.

Nitrogen adsorption experiments were performed on a Micromeritics ASAP 2010 porosimeter at 77 K (Micromeritics Instrument Corporation, Norcross, GA, USA). Samples were degassed to 1 μm Hg at 100 °C prior to analysis. Surface area was determined by use of the Brunauer-Emmett-Teller (BET) method, pore size was calculated by the Barrett-Joyner-Halenda (BJH) method from the adsorption branch of the isotherm, and total pore volume was determined by the single point method at relative pressure (P/P_0_) 0.97. Scanning electron microscopy (SEM) was performed with a LEO 1455 SEM (Carl Zeiss SMT, Inc., Peabody, MA, USA). A tungsten filament and secondary electron detector were used at a beam voltage of 15-20 kV. Samples were mounted on SEM stubs using conductive carbon tape and sputter coated with gold or palladium using a Cressington 108 auto sputter coater for 60 s. Thermogravimetric analysis (TGA) was performed with a TA Instruments Hi-Res 2950 thermogravimetric analyzer (TA Instruments, Inc., New Castle, DE, USA). The temperature was ramped 1 °C/min to 400 °C under a nitrogen atmosphere.

## 3. Results and Discussion

### 3.1. Sorbents for Nitroenergetics

**Figure 1.**
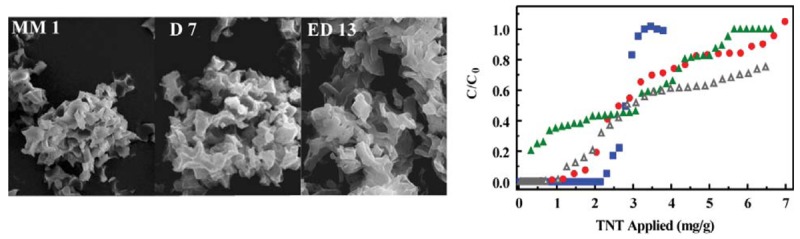
Impact of sorbent variations. (**A**) SEM images of MM1, D7, and ED13 showing variations in macro-morphology. (**B**) TNT breakthrough curves for MM1 (blue squares; 200 mg sorbent), D7 (red circles; 82 mg sorbent), and ED13 (green triangles; 141 mg sorbent). Data for ED13 at a lower flow rate (gray triangles) is also presented.

Our initial reports on diethylbenzene bridged organosilicate sorbents focused on determining precursor ratios providing a balance between morphological and binding characteristics and on development of semi-selective binding characteristics within the sorbents [14,23,24]. More recently, we presented proof of concept data demonstrating the function of those materials in relevant matrices [25]. Since completion of those studies, we have attempted to improve the performance of the sorbents through further alteration of chemical group content and morphological characteristics [24]. Several of the subsequently developed materials have been evaluated to determine what, if any, advantages they offer over the originally described MM1 sorbent. MM1 was synthesized using a 1 to 1 ratio of diethylbenzene (DEB) bridging groups to ethane (BTME) bridging groups with 12.6% of the total surfactant consisting of a modified surfactant imprint template (Table 1) [23]. The material offers macroscale texture with an ordered mesopore structure. The macroscale texture provided reduced the pressure needed to drive fluid flow when sorbents were applied in column formats for solid phase extraction. The subsequently developed ED13 was synthesized with increased concentrations of acid and swelling agent [24]. The result was a more defined macroscale texture (Figure 1) as well as increased surface area, pore volume, and pore diameter (Table 1). D7 was synthesized using 100% DEB bridging groups with further modified acid and swelling agent concentrations [24]. DEB groups were initially found to provide higher binding capacity for nitroenergetic targets at the expense of morphological characteristics and selectivity [22]. The synthetic protocol developed for D7 provided morphological characteristics similar to MM1.

The performance of the newly developed materials was compared to that of MM1 (Figure 1) using column format, target breakthrough testing. While D7 provided a total TNT binding capacity similar to MM1 (Table 1), the breakthrough profile showed early initial target breakthrough with total capacity reached through slow partial target binding. MM1, on the other hand, initially bound all applied target reaching a sharp breakthrough much closer to the total binding capacity. ED13 provided an improved TNT binding capacity; however, the breakthrough characteristics of this sorbent were similar to those of D7. The increased binding capacity was likely due to the increased available surface area (Table 1) and improved accessibility of that surface area due to larger mesopore diameters. ED13 also offered less resistance to flow of solutions through the sorbent column than either the D7 or MM1 sorbents. This reduced resistance contributed to the observed breakthrough characteristics as contact time with the sorbent was reduced under an equivalent driving pressure. When the flow rate through ED13 (initially 2.5 mL/min) was reduced to match that achieved with MM1 and D7 (1 mL/min), an extended region of complete target binding was obtained (Figure 1).

Though the earlier initial breakthrough observed for ED13 would appear to make MM1 the favored material, there are other considerations in selection of the best sorbent for the applications of interest here. First, the issue of backpressure is a critical consideration when forcing liquids through the column. The goal, here, is to develop a portable system for target preconcentration, so power requirements are an important consideration. Greater pressure in the system requires a larger driving force and, therefore, greater energy consumption. This aspect and the advantages of the ED13 sorbent over MM1 are discussed in detail in the selection of system components in the section below. Initial breakthrough for the ED13 column occurred at 0.74 mg/g. At the drinking water equivalent level (DWEL) of 20 μg/L for TNT, a system using a 100 mg sorbent column could bind 100% of the target from 3.7 L of water before reaching the breakthrough loading level. This volume is well beyond the sample volume intended for the application under consideration. Sampling volumes of less than 0.1 L are expected. Given these considerations, ED13 offers a significant advantage over MM1 in the reduction of backpressure.

### 3.2. Liquid/Liquid Preconcentration

**Figure 2.**
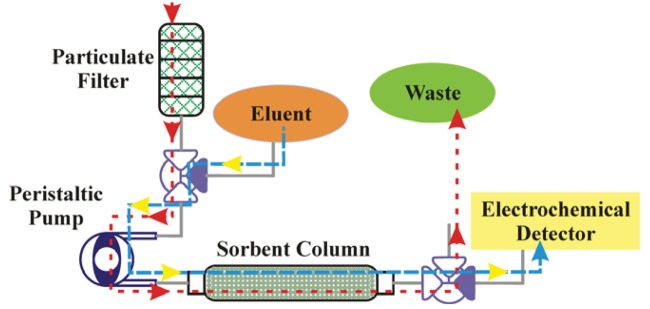
Schematic of the liquid/liquid system. Here, the red line indicates the flow of the sample solution through the system, and the blue line indicates the flow of eluent through the system.

Having developed novel sorbents providing binding characteristics necessary for preconcentration, it was necessary to have the applicable associated systems. Figure 2 presents a schematic of the system concept for use of the sorbents with aqueous samples and liquid target elution. The system was intended to provide sample collection, preconcentration, and elution prior to analysis by an electrochemical detector. Here, aqueous samples are pulled through a filter stack to eliminate particulate (size exclusion). A peristaltic pump is employed to pull the sample through filters and a 3-way solenoid valve and to push it through the column where target is captured by the sorbent. A peristaltic pump is desirable as the liquid does not come in contact with the pump machinery and, therefore, issues related to corrosion and clogging are minimized. The use of a single pump also provides the potential for a more compact final system. The post column solenoid valve ensures that the sample that has passed through the column is discarded. Once target is adsorbed to the sorbent, the pre-column valve is switched and eluent is pushed through the column by the peristaltic pump. The desorbed targets are then directed to the electrochemical detector by switching the post-column solenoid valve. In order to evaluate this approach to design of a system, a bench scale, bread-board level, prototype was assembled using a peristaltic pump with a 900:1 motor and 0.143″ rollers (P625/900.143, Instech Laboratories, Plymouth Meeting, PA, USA). The pump was combined with 0.031″ silicone tubing to provide flow rates up to 0.8 mL/min through the sorbent column using a 9 V battery. The operation voltage for the solenoid valves (5 V, Lee Products, Westbrook, CT, USA) is controlled using on/off switches. Sorbent columns (MM1) were prepared in stainless steel HPLC column housings (4.6 × 33 mm, Bischoff Chromatography, Leonburg, Germany) using a mass of 60 mg.

**Figure 3.**
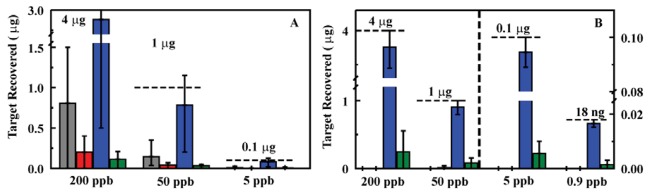
Analysis of samples using the liquid/liquid system with an HPLC column housing (60 mg, MM1 sorbent). Flow rates varied between 1.0 and 0.3 mL/min. Adsorption of target (effluent; gray) was followed by a water rinse (3 mL; red), target elution (2 mL acetonitrile; blue), and an additional water rinse (3 mL; green). (**A**) TNT experiments for which there was tunneling through the sorbent column. (**B**) TNT experiments for which the flow rate was slowed to 0.3 mL/min by the excessive pressure in the system. All data points are the average of three target adsorption/elution cycles. Similar results were obtained for RDX, HMX, and DNT (not shown).

A series of experiments using nitroenergetic targets in deionized water was completed using the prototype system. Sample solutions containing target concentrations of 200, 50, 5, and 0.9 ppb for TNT, RDX, DNT, NG, and HMX were evaluated in triplicate. For each sample, 20 mL of the sample solution was passed through the sorbent. A water wash of 3 mL was then passed through the column followed by 2 mL acetonitrile and an additional 3 mL water wash. Each of the volumes was analyzed by HPLC to determine target retention and the preconcentration potential of the system (Figure 3). These experiments yielded highly variable results. We found that, though the system performed for five to eight samples, column pressures increased with successive usage cycles. The result of increasing back pressure was a reduction in flow rate (0.8 mL/min to as little as 0.2 mL/min) and tailing of the eluted target into the final rinse volume. Alternatively, tunneling through the sorbent (passing of solutions through voids in the column rather than through pores in the sorbent) resulted, leading to poor target retention. Tunneling was observed as failure of the column to bind the targets and higher than expected flow rates (>0.8 mL/min). The frits provided with the column housings were also highly subject to clogging both by particluate in the samples and by sorbent material.

**Figure 4.**
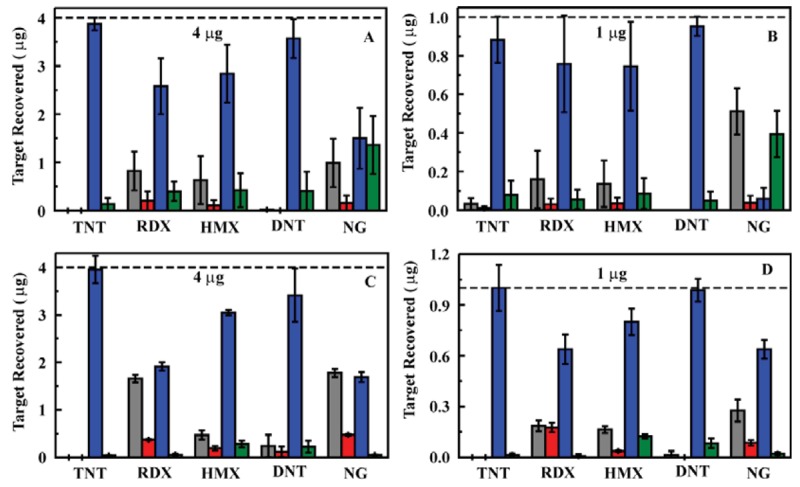
Analysis of samples using the liquid/liquid system with a 10 mm Omnifit column housing and 200 mg ED13 sorbent. Targets were applied at 0.8 mL/min. Adsorption of target (effluent; gray) was followed by a water rinse (3 mL; red), target elution (2 mL acetonitrile; blue), and an additional water rinse (3 mL; green). All data points are the average of three target adsorption/elution cycles. Samples were prepared in deionized (200 ppb (**A**) and 50 ppb (**B**)) and well water (213 m well; 200 ppb (**C**) and 50 ppb (**D**)).

The column housing was changed in an attempt to improve flow rates and reduce pressure while providing the desired target retention. An Omnifit borosilicate column housing (6.6 × 50 mm, Diba Industries, Mahopak, NY, USA) was selected, and a 75 mg sorbent column (MM1) was packed. The experiments described above were repeated using this sorbent column in the prototype system with somewhat improved results; however, the performance still fell short of what was expected based on previous experiments [23,25]. Both the increased pressure and tunneling effect observed for the HPLC column housing were observed for this housing as well. Based on the reduced pressures observed for the ED13 sorbent (Section 2.1), this sorbent was substituted for MM1 in the experiment. This led to a significant improvement in the pressure in the system over repeated tests. Further improvement was achieved through replacement of the column housing with one of a larger diameter (10 mm) allowing for an increase in the sorbent mass utilized (200 mg; Figure 4). The results achived with the prototype system were comparable to those observed for the sorbents when used in an offline configuration [23,25].

**Figure 5.**
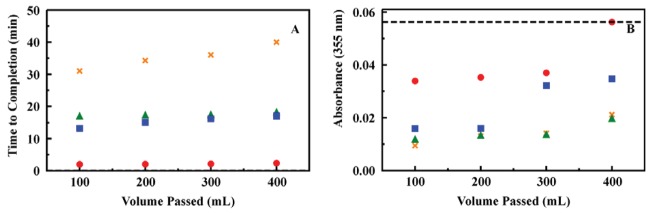
Filtration of particulate using Whatman GF/F and GF/D filters. The pressure generated by the filter configurations was determined based on the time to complete passage of 100 mL with no additional applied pressure (**Panel A**). The filtration setup used a Chemglass beaker with glass frit (40 μm pore size). A Millipore dual opening funnel was clamped to the beaker. The filter configuration was placed between the funnel and the beaker frit. The solution to be filtered was prepared by addition of fine soil to deionized water (5 g/L). The effectiveness of filtration was determined from turbidity based on the absorbance intensity of the solution at 355 nm (**Panel B**). GF/D (red); GF/F (blue); stack of two GF/F filters (orange); stack of one GF/D and one GF/F filter (green); dashed line indicates turbidity of initial solution.

A sensing system of this type requires filters to protect the column from particulate. Combinations of standard filters were evaluated to determine which would provide the necessary level of particulate removal without nonspecific target capture. Given the pressure issues noted above, filters providing minimal increase in pressure were also desired. Whatman GF/F (0.7 μm pores) and GF/D (2.7 μm pores) glass fiber filters were selected. Nonspecific binding of targets to these types of filters is negligible. Combinations of these filters were evaluated, and a two filter series (GF/D followed by GF/F) was found to provide the necessary protection for the system components (Figure 5).

**Figure 6.**
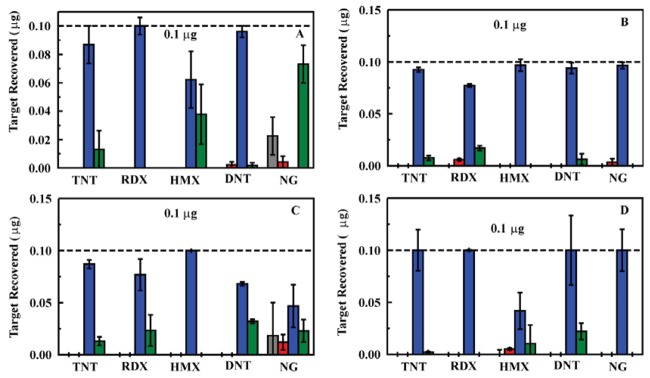
Analysis of samples in varied matrices. Here, the liquid/liquid system was used with a 10 mm Omnifit column housing and 200 mg ED13 sorbent. Targets were applied at 0.8 mL/min. Adsorption of target (effluent; gray) was followed by a water rinse (3 mL; red), target elution (2 mL acetonitrile; blue), and an additional water rinse (3 mL; green). All data points are the average of three target adsorption/elution cycles. Samples (5 ppb) were prepared in deionized water (**A**), 114 m well water (**B**), 122 m well water (**C**), and 213 m well water (**D**).

Having established a prototype system that would function as desired, a number of samples were evaluated to provide an indication of performance over an extended use period. A single sorbent column (the same used for samples in deionized water, 200 mg ED13) was utilized for evaluation of spiked ground water samples (Figures 5 and 6). Ground water samples were collected from household wells in Clarksville, MD (depth of 114 m) and Fulton, MD (depths of 122 and 213 m), USA to provide matrices of varying composition. The well at 213 m provides water that smells of sulfur while water from the other wells was free of odor. Water from the well at 213 m appeared slightly cloudy while water from the other wells was clear. (Filtration prior to analysis is described below) In total, 175 samples were evaluated using the column with 75 of those in ground water (15 samples at 200 ppb, 15 samples at 50 ppb, and 45 samples at 5 ppb). Each sample consisted of application of the target solution (20 mL) followed by rinsing of the column with deionized water (3 mL). The target was then eluted in acetonitrile (2 mL) and the column was again rinsed with water (3 mL) to prepare it for the next sample. All four of these volumes were analyzed individually by HPLC to determine target content and concentration.

The total time for the solid phase extraction processing using the 200 mg ED13 column in the prototype system was 30 min. This time requirement is dependent on the flow rate of solutions through the system (1 mL/min in this case). In all cases, target was detected in the spiked ground water samples. The enhancement in target concentration in the eluent volume varied from target to target as expected based on previous results [25]. There was some variation between the matrices as well. The pH of all of the water samples was between 6 and 6.5. Since all samples were filtered prior to validation by HPLC or passage through the prototype, the particulate levels were the same. Ion chromatography (EPA Method 314) indicated varied levels of sulfate and nitrate in the samples, with the 213 m well showing the highest concentrations as well as smelling of sulfur. We have reported similar variations in enhancement for experiments in which samples were prepared in artificial sea water or for targets extracted from soil samples [25]. Though the factors influencing the differences have not been thoroughly identified, the variations are not unexpected in complex sample matrices.

During analysis of this sample series, it was necessary to replace the silicon tubing used in the peristaltic pump three times. Continued use of a single tubing set resulted in damage and eventual rupture of the tubing after 100 to 150 h of system use. Reduction in the pressure within the system is necessary to stabilize the pump and prevent this damage. The pressure in the prototype system is overwhelmingly due to the sorbent column. We found that a significant portion of the pressure generated by the column could be reduced through changing the frits used. The frits supplied with the Omnifit columns are PTFE with 10 μm pores. Use of 25 μm PTFE frits reduced the pressure in the system (as evidenced by greater flow rates under identical driving pressure). We are currently investigating alternatives to the PTFE frits that may allow further pressure reductions. Should further reduction of pressure in the column not be possible, alternative pumps such as miniature hydraulic or external gear pumps may be necessary for extending the use time of such a system between maintenance.

### 3.3. Liquid/Vapor Preconcentration

**Figure 7.**
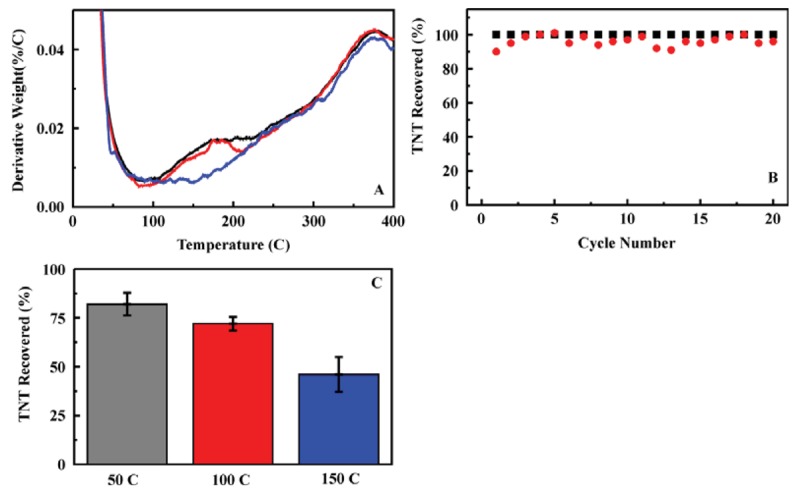
Thermal cycling and desorption. (**A**) Thermal gravimetric analysis of ED13 sorbent (25 mg, blue) loaded in batch adsorption experiments with TNT (black) and RDX (red) (50 μg). Derivative weight is plotted *versus* temperature to provide better visualization of the differences between the data sets. (**B**) The performance of the sorbent was not degraded over 20 heat/cool cycles. Each cycle was 45 mins at 150 °C. The total percent target bound (from 10 mL of a 200 ppb TNT solution) and recovered (2 mL acetonitrile elution) is presented for each cycle. (**C**) Shown here is the total percent of the target recovered (column loaded with 10 mL of a 200 ppb TNT solution) following flow of air through the sorbent column at the indicated temperature (30 mL, 3 mL/min).

Ion mobility spectrometry (IMS) can be applied to detection of targets in vapor phase. Applications of polymers to the capture and preconcentration of targets prior to IMS analysis have been described in the literature. Often, those polymer materials are susceptible to swelling when exposed to increasing humidity levels [30,31]. The sorbents described above offer a possible alternative to polymer materials providing adsorption of the target from aqueous solution followed by thermal desorption into a vapor phase. The sorbents are not subject to swelling or pore collapse under changing humidity. In order to evaluate their potential in this application, thermogravimetric analysis of the sorbents was used to evaluate desorption of TNT and RDX from the sorbent. Target desorption was noted over the temperature range between 100 and 210 °C (Figure 7). It was also necessary to determine if the sorbents were stable over repeated thermal cycling. For this determination, a sorbent column, ED13 in an HPLC housing, was utilized repeatedly for TNT adsorption followed by thermal cycling to 150 °C (45 min) and target elution. No performance degradation was observed for a sorbent column utilized over 20 cycles (Figure 7).

On the basis of the stability of the sorbent and the potential for target desorption, thermal desorption from a sorbent column format was evaluated. TNT was loaded onto the column (10 mL, 200 ppb) and a water wash (3 mL) was performed, following the protocol used for the liquid/liquid system (Section 2.2). Air was then passed through the column (30 mL) to remove residual liquid from the sorbent material. The sorbent column was heated (in a calibrated laboratory oven) for 20 min to allow stabilization at temperatures ranging from 50 to 150 °C. Heated air (30 mL) was then pushed through the column at a flow rate of 3 mL/min. Residual target was eluted (2 mL acetonitrile) and the sorbent was rinsed to prepare for the next sample cycle. Each of the volumes (effluent, washes, and eluent) were analyzed by HPLC to determine target content and concentrations. A significant reduction in the target recovered in the eluent was noted for sorbent columns subjected to heating and air purge with a distinct dependence on the column temperature (Figure 7). The reduction in target recovered was attributed to the percentage thermally desorbed during the air flow step. Reported limits of detection for portable IMS systems, such as the SABRE 4000 (Smiths Detection), are in the picogram range. Desorption of 25% of the adsorbed target at 100 °C (Figure 7) far exceeds this limit of detection. The results presented in Figure 7 are proof of concept only. There are other factors such as humidity and target delivery to be considered for use of a preconcentration system with IMS. The results do, however, indicate the potential for sorbent application in thermal desorption and provide motivation for proceeding to system design.

**Figure 8.**
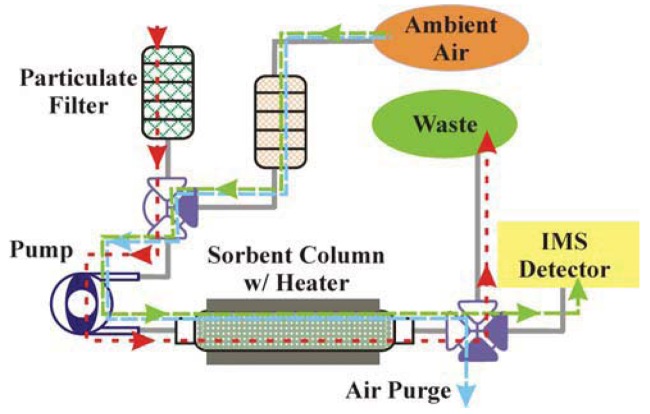
Schematic of the liquid/vapor system. A single pump is used to provide the driving force for movement of solutions and air through the system with control provided by switchable valves. Filters are used to protect the system from particulate in both the ambient air and in the water samples. Here, the red line indicates the flow of the sample solution through the system, the blue line indicates the flow of purge air through the system, and the yellow line indicates the flow of air for target desorption through the system.

Figure 8 presents a schematic of the liquid/vapor system concept. The system would provide sample collection, preconcentration, and elution as a vapor prior to analysis by a commercial IMS detector. This system uses a similar approach to the liquid/liquid system described above. Aqueous samples are pulled through a filter stack, and a single pump is employed to move all volumes. Once target is adsorbed to the sorbent, the column is heated and the desorbed targets are directed to the IMS. We are currently selecting components for assembly of this system and trying to optimize the conditions for thermal desorption including temperature, temperature ramp-rate, and air flow rates and volumes.

## 4. Conclusions

Here, we have described our ongoing effort related to providing materials and systems that offer improved detection of nitroenergetic water contamination with a view toward offering *in situ* water quality monitoring. The ED13 sorbent described offers increased nitroenergetic sorption capacity over our previously evaluated sorbent (MM1) [23,25] while also providing reduced back pressure in the column format. This reduced pressure is important for development of a portable system due to power requirements and component durability. The liquid/liquid preconcentration system described here is an early prototype; however, it demonstrates the potential for application of the sorbents to inline preconcentration and detection. Even in the form utilized, continuous analysis of samples over the course of several weeks was possible with minimal maintenance. Improved performance may be obtained through the use of alternative pumps (*i.e.*, external gear pumps) providing more rapid flow through the sorbent column. In addition, initial experiments indicate that thermal treatment is a possible method for target desorption. This may offer an avenue for combining target extraction by the sorbents with IMS detection by a commercially available portable instrument, though exact conditions and components for thermal desorption of targets have not been optimized. We hope to evaluate a prototype inline system utilizing thermal desorption with a SABRE 4000 (Smiths Detection) in the near future. The two options presented here, liquid and vapor target delivery, should provide flexibility for addressing a wide range of monitoring needs.

## Figures and Tables

**Table 1.** Material composition and characteristics.

**Material**	**Composition**	**Mass TMB (g)^1^**	**Mass HNO_3_(g)^2^**	**Surface Area (m^2^/g)**	**Pore Vol (cm^3^/g)**	**Pore Dia (Å)**	**Sorbent Mass (mg)^3^**	**TNT Capacity (mg/g)**	**Initial TNT breakthrough (mg/g)**
MM1 [23]	50:50 BTE:DEB	0.20	6.0	366	0.26	35	200	2.7	2.1
ED13 [24]	50:50 BTE:DEB	0.60	7.5	712	0.75	65	141	3.2	0.3 4
D7 [24]	100 DEB	0.90	7.5	414	0.42	49	82	3.1	0.4

1 Mass of TMB included with 1.9 g total surfactant;

2 Mass of 0.1 M HNO_3_ solution included in synthesis;

3 Sorbent mass utilized for column breakthrough experiment;

4 Reduction of flow rate to 1 mL/min increases the value to 0.74 mg/g.
